# The Social, Economic and Sanitary Impact of COVID-19 Pandemic

**DOI:** 10.1590/S1677-5538.IBJU.2020.S1ED2

**Published:** 2020-07-27

**Authors:** Ana María Autrán Gómez, Luciano A. Favorito

**Affiliations:** 1 University Hospital Fundación Jiménez Díaz Department of Urology Madrid Spain Department of Urology, University Hospital Fundación Jiménez Díaz, Madrid, Spain; 2 Universidade do Estado de Rio de Janeiro - UERJ Unidade de Pesquisa Urogenital Rio de JaneiroRJ Brasil Unidade de Pesquisa Urogenital da Universidade do Estado de Rio de Janeiro - UERJ, Rio de Janeiro, RJ, Brasil

## COMMENT

In the last 3 months, nearly a third of the world's population has changed their lifestyle. At this time of writing (June 30th) the COVID-19 pandemic has left a total of 10.302.867 confirmed cases, with more than 505.518 deaths worldwide, spreading to more than 188 countries ( 1 ). The pandemic has hit each of the different social strata, the population has had to re-adapt to the circumstances, absolutely all of us have changed the way we face the day to day.

Asia, Europe and recently Latin America have been involved in this catastrophe, where the delay in the implementation of health policies by governments has aggravated the problem.

Currently, Brazil, one of the main countries and economic force in Latin America, occupies the second place in the world with a total of 1.368.195 confirmed cases and 58.314 deaths ( 1 ).

The COVID-19 pandemic represents a sanitary, social and economic challenge at a global level, since the City of Wuhan in China declared it's lockdown on January 23th 2020, about a third of the world's population has had to follow the same policies of restriction and isolation at home, imposed by governments, to reduce the spread of the disease to avoid the collapse the health system, measures that today are not entirely clear, as they have been implemented as “emergencies”. This is the result of a lack of adequate epidemiological strategies, focused on the impact of the dissemination of the disease, with the application of tests to the population, tests that we do not currently have. Lockdown measures have led to the cessation of industrial and commercial production in most sectors, with job reductions and layoffs. Recently an editorial in the newspaper The Economist ( 2 ) revealed that approximately the cessation of economic activity produced by the lockdown and reduction of movement, will lead a total of 420,000,000 people to absolute poverty with incomes of less than USA $1.90 a day. If we look at first world countries, the United States (USA) has reported a gradual increase of 3.5% in unemployment in February versus 14.7% in April 2020.

The COVID-19 is without a doubt one of many pandemics that humanity has had to face throughout history. Never before has the fear of death been so pronounce, because we are reminded daily that people are dying in alarming numbers of around 100 deaths per minute and 150,000 deaths per day. This fear of loss, coupled with social distancing, lockdown, economic instability and uncertainty, will result in a strong psychosocial impact that will have to be addressed ( 3 ).

Regarding to international Health Systems, the impact on the cessation of development and stabilization in the coming months is enormous. It is estimated that England, by prioritizing care for COVID-19 patients and reducing care for cancer patients, within 6 months, will lose 40% of the quality of life gained in the last 5 years. The World Health Organization warns that if, as a result of the health impact of the pandemic, vaccination programs are paralyzed, around 140 children in Africa will die from each death caused by COVID-19. Just three months into lockdown, 10 months of discontinuation of Tuberculosis treatment in Third World countries will follow, equivalent to approximately 1,4000,000 deaths between 2020-2025.

Therefore, health systems require an adequate cost-benefit balance between the health policies and economic resources established in each country to face the pandemic, which must be directed on the basis of social risk groups, development dynamics, developmental heterogeneity and resources.

In most countries, the contingency and reaction plans established by the government have been misdirected, at the wrong time, implementing urgent measures, most of them with little effectiveness, we have faced a shortage of resources, lack of medical equipment, exhaustion with psychological burn out, infections of health care workers, how many of us have not suffered the disease and have seen so many colleagues infected?. Only in Spain the latest publication of the Spanish Ministry of Health ( 4 ) (June 12th 2020), has reported a total of 51. 849 infected health care workers.

Regarding the management of assistance protocols and triage, they have been constantly modified in each centre, in each province, trying to adapt to the resources and assistance demands of each country.

Without a doubt, all these experiences have enriched our knowledge, survival and management of the COVID-19 pandemic in each of our countries.

The volume of publications and scientific articles that have been written and made available is impressive. The medical community worldwide has joined efforts to be able to transmit quickly and effectively a large number of constant information from different medical specialties. The social networks have been overturned with the diffusion of information, which on some occasions, we could call misinformation.

In this context, urology, without a doubt, has not been the exception as since the end of March 2020, different International Urological Societies, have added their efforts to be able to establish recommendations, which have allowed us to optimize and prioritize patients' care being implemented, in most Urology services. We have learned that patient care can continue, through the introduction of Telemedicine, that medical education is feasible and that we can share knowledge and with residents to continue their training.

At this time, we have shared academic sessions with our colleagues and friends around the world thus discovering these new tools of communication and development. We have also learned that we can grow among all, expanding our network of scientific collaboration, all these leaves us the COVID-19 Pandemic

As part of an international collaborative effort, the American Confederation of Urology (CAU) and Sociedade Brasileira de Urologia (SBU) performs this special edition, which aims to provide a screenshot impact of the COVID-19 Pandemic on Urology, within each different area of development It's contains a total of 27 manuscripts performed by expert urologists from France, Italy, Spain, Iran, Germany, Argentina, Uruguay, Brazil, Mexico, Peru, Bolivia, Panama, the USA. The information that it contains, is reported until May 8th 2020.

Finally, I would like to thank each colleague participating in this project, for the effort and valuable academic contribution, hoping that this crisis we are going through will allow us to grow as people and professionals. “Every crisis has a solution and a learning process, this only depends on us”
